# Notices

**Published:** 1866-06

**Authors:** 


### NOTICES.

Our Daughter's Schooling.—The sisters Bucknall have retired from the more arduous labors of a large school for young ladies in New York city, and have removed to their beautiful country seat near New Brunswick; where, not abandoning a field altogether, in which for so many years they successfully labored, they will still continue to give instruction to a select few; this will be interesting intelligence to their patrons and scholars, which latter, after entering married life, have repeatedly come to their former teachers for the express purpose of assuring them how much they appreciated their fidelity and conscientious and untiring efforts to make their moral and literary education what it ought to be, and which they more highly valued now, than when they stood in the relation of pupils and teachers; this simple fact of itself tells volumes in just praise of these admirable and able instructors of so many of the daughters of New York.

The Boston American Tract Society, fully alive to the importance of the subject, have issued "The Freedman's Reader" and "The Freedman's Spelling Book" which are admirably adapted to the purpose for which they were intended. Also the "Beloved Disciple," by J. W. Kimball, treating of Faith, Love, Purity, Gravity, Humility, Courage, Honesty, Benevolence and other practical christian virtues; these books are to be had at 28 Cornhill, Boston and at 13 Bible House, New York city.

Every Saturday.—This Weekly is, in our opinion, precisely what it claims to be,—a journal of *choice* reading selected from current literature. The editor has the range of all the English and Continental Reviews, Magazines, and first-class Weeklies, which press into their service the ablest, wisest, and wittiest writers of Europe. From this almost immense storehouse, he selects that which he judges best adapted to suit the taste and intelligence of the American people.

The selections in the numbers already issued have embraced a wide variety of topics,—all of interest to cultivated minds, and nearly all of a character to be highly attractive to the majority of American readers. There have been excellent short stories, thrilling adventures, exquisite poems, graphic historical sketches, popular scientific articles such as appear originally only in English and French periodicals, racy essays in biography, criticism, and anecdote. In fact, it contains the cream of foreign current literature, and is offered at a reasonable price.

Each number being complete in itself, it is just the thing for travellers; and each number is of such sterling merit that it is just the thing for those who stay at home. Whoever wishes the freshest and choicest foreign periodical literature, must get "Every Saturday." It is published by Ticknore & Fields, Boston.

S. V. S. Wilder, The Life of, 404 pp. 12 mo., published by the American Tract Society, 150 Nassau St., New-York. Mr. Wilder was born in Lancaster, Mass., in 1780, and died in 1865, in the bosom of his family, surrounded by children and grand-children. It is the record of a good man, who in all his intercourse with men of distinction and influence at home and abroad, never failed to let his light so shine, that all took knowledge of him that he was a christian. He was intimately associated with such men as Fulton, Madison, Monroe, Jackson, Morse, and others; the book is full of incidents of life in France, Paris, court life, &c.; it is highly instructive, and is well worthy of a very general perusal; in many portions of it, it is stranger than fiction.

THE BANKERS OF THE WORLD.—" The Merchants and Bankers' Almanac for 1866," one volume octavo, pubished at the Bankers' Magazine office, N. Y., contains lists of 1620 National Banks, (with names of the President and Cashier and N. Y. Correspondent of each), 400 State Banks ; 1100 Private Bankers in the U. S. ; Banks and Bankers in London, Liverpool, Dublin, Edinburgh, Leeds, Manchester, Birmingham, &c. ; 600 Bankers in Europe, Asia, Africa, Australia, the West Indies, South America, New Zealand, Mexico, Canada, &c. ; Alphabetical list of 2000 Cashiers in the U. S. ; list of 300 Savings Banks in New England and New York, with the deposits of each ; Bank Statistics of the U. S. ; list of Standard Works for Bankers ; prices of Iron, Copper, Coal, monthly at N. Y. 40 years ; Daily price of Gold for four years, 1862-1865 ; and six engravings, viz. : 1. The New York Stock Exchange, (erected 1865) ; 2. The Paris Stock Exchange, (1808-1826) ; 3. The Bank of England ; 4. Banking Houses, Wall Street ; 5. New Insurance Buildings, Broadway, N. Y. ; 6. The Mutual Life Insurance Company of N. Y. Price $2.00

'Stonewall Jackson," A minute account of his last moments and death, by Hunter McGuire, M. D., Prof. of Surgery, and Medical Director &c. It seems that General Jacksons' death resulted, not from wounds received in battle, but from having fallen while being carried from the field. His last words were, "Let us cross over the river and rest under the shade of the trees." He refused to take brandy. " I want to preserve my mind, if possible, to the last." The whole narration is intensly interesting, as found in the Richmond, Va., Medical Journal for May, 1866, $5 a year, single Nos. post-paid, Fifty cents; this number contains also interesting articles on Hip Deformities, Progress of Surgery, Gun-shot Wounds, Military Hospital experiences &c.; it is a most valuable number.

DEFORMITIES and blemishes of the face, eye, nose &c. are treated with singular skill and success by Dr. Daniels at his rooms on Union Square, New York city, corner of 14th St. & Fourth Avenue, as also all ailments requiring the clear sight and steady hand of a practical surgeon.

ONE THOUSAND ACRES of fertile land, with a dwelling, in Cumberland Co., East Tennessee, on the direct road from Nashville to Knoxville and from Cincinnati to Chattanooga and Charleston, S. C. can be purchased for Five Thousand dollars cash ; part timbered, part prairie ; or would be exchanged for productive real estate in New York city or Brooklyn, apply to P. C. Godfrey, 823 Broadway, New York.

COMPARATIVE PHYSIOGNOMY, or resemblence between man and animals, by James W. Redfield, M. D., 330 engravings, W. J. Widdleton, New York Publisher. The New York Observer of May 17th, says : " The reader cannot fail to be entertained with this book, aiming to show that there are strong points of facial resemblence between men and animals, and that this outward resemblence is indicative of a character similar to that of the animal whose likeness is seen in the human face. The argument is not less ingenious than the illustration, and its aim seems to be to discredit phrenology, and establish physiognomy as a more reliable index of character. Sent post-paid for Three Dollars.

